# Stromal Keratitis among Herpes Simplex Keratitis Patients in a Tertiary Eye Hospital: A Descriptive Cross-sectional Study

**DOI:** 10.31729/jnma.7906

**Published:** 2022-12-31

**Authors:** Poonam Shrestha, Santosh Paudel

**Affiliations:** 1 Department of Cornea and External Eye Disease, Nepal Eye Hospital, Tripureshwor, Kathmandu, Nepal; 2 Nepal Eye Hospital, Tripureshwor, Kathmandu, Nepal

**Keywords:** *herpes simplex keratitis*, *keratitis*, *prevalence*

## Abstract

**Introduction:**

The manifestations of herpes simplex virus keratitis range from epithelial keratitis to vision-threatening stromal keratitis. There are limited studies done on our part regarding stromal keratitis. The aim of the study was to find out the prevalence of stromal keratitis among herpes simplex keratitis patients in a tertiary eye hospital.

**Methods:**

This descriptive cross-sectional study was conducted among patients who presented with herpes simplex virus keratitis in a tertiary eye hospital between 1 January 2020 to 28 February 2022. Ethical clearance was taken from Ethical Review Board (Reference number: 1/2079/80). Data was collected from hospital records which was reviewed and visual acuity at presentation and at one-month follow-up, clinical details on examination were recorded and, diagnosis of the stage of disease given in the case record was noted. Convenience sampling was used. Point estimate and 95% Confidence Interval were calculated.

**Results:**

Among 112 Herpes simplex keratitis patients, the prevalence of stromal keratitis was 38 (33.93%) (25.16-42.70, 95% Confidence Interval).

**Conclusions:**

The prevalence of stromal keratitis among patients of herpes simplex keratitis was similar to studies conducted in similar settings.

## INTRODUCTION

Herpes simplex virus (HSV) is a ubiquitous DNA virus that can infect virtually anywhere in the body however, the most common site of infection are the mouth, genitalia and eye.^1^ Herpetic stromal keratitis (HSK) comprises three major subtypes: epithelial, stromal, and endothelial.^2^ Stromal keratitis develops as a result of an immune response to the virus. The stromal subtype can be further divided into disciform keratitis, immune stromal keratitis, and necrotizing keratitis.^3^

HSV stromal keratitis is a very common infective disease in developed countries and it has been estimated that nearly 500,000 people in the USA are affected with ocular HSV.^4^ The frequency of incidence of viral stromal keratitis has become much greater in developing countries also because of improved socioeconomic status and following indiscriminate widespread use of antibiotics and corticosteroids.^5^ The epidemiology of ocular involvement with HSV has not been well defined in developing countries despite its high prevalence in developed countries.

The aim of the study was to find out the prevalence of stromal keratitis among Herpes simplex keratitis patients in a tertiary eye hospital.

## METHODS

A descriptive cross-sectional study was conducted among patients who presented with herpes simplex virus keratitis in Nepal Eye Hospital between 1 January 2020 to 28 February 2022. Ethical clearance was taken from the Institutional Review Board (IRB) of the National Academy of Health Science (Reference number: 1/2079/80). Data was collected from hospital records where visual acuity at presentation and clinical details on slit-lamp examination were recorded and diagnosis of the stage of disease and visual acuity at 1 month follow-up was also noted. All cases of herpes simplex keratitis patients visiting to Nepal eye hospital and giving consent were included and those patients who have herpes simplex keratitis with epithelial and endothelial keratitis were excluded. Convenience sampling was used. Sample size calculated by using the following formula:


$$\begin{array}{ll} \text{n} & ={\text{Z}}^{2}\times \frac{\text{p}\times \text{q}}{{\text{e}}^{\text{2}}}\\ & =1.96^{2}\times \frac{0.50\times 0.50}{0.10^{2}}\\ & =97 \end{array}$$

Where,

n = minimum required sample size
Z = 1.96 at 95% of Confidence Interval (CI)
p = prevalence is taken as 50% for maximum sample size calculation
q = 1-p
e = margin of error, 10%

Stromal keratitis included cases of immune stromal keratitis, which can manifest as focal, multiple, or diffuse stromal opacities often accompanied by oedema, and cases of interstitial keratitis, which have accompanying blood vessels with stromal opacity.^6^

Data were entered and analysed with IBM SPSS Statistics version 25.0. Point estimate and 95% CI were calculated.

## RESULTS

Among 112 herpes simplex keratitis patients 38 (33.93%) (25.16-42.70, 95% CI) cases presented with purely stromal lesions. The age of stromal keratitis patients ranged from 10-73 years, with a mean age of 47.19±19.14 years. Among the total 38 cases of stromal keratitis, 28 (73.68%) were female and 10 (26.31%) were male. Most of the patients had a history of spontaneous onset of disease followed by other triggering factors (Table 1).

**Table 1:** Triggering factors for disease (n= 38).

Triggering factor	n (%)
Spontaneous	19 (50.00)
Stress	10 (26.32)
Fever	13 (34.21)
Trauma	8 (21.05)
Foreign body	6 (15.79)
Skin eruptions	6 (15.79)
UV light	5 (13.16)

The common symptoms were mild pain, redness, decreased vision, photophobia, and watery discharge out of which 22 (19.6%) patients presented with mild pain, 90 (80.35%) with redness, 49 (43.75%) photophobia, 53 (47.32%) decreased vision and 44 (39.28%) watery discharge. The visual acuity of all stromal keratitis cases was recorded (Table 2).

**Table 2:** Presenting visual acuity of stromal HSV keratitis patients (n= 38)

Visual acuity at presentation	n (%)
6/6-6/18	5 (13.16)
6/24-6/60	12 (31.58)
<6/60-3/60	13 (34.21)
<3/60	8 (21.05)

Twenty-seven (71.05%) patients had no previous attack of the disease, whereas 11 (28.9%) had a history of recurrence. Among 38 patients of stromal keratitis 31 (81.5%) patients had unilateral presentation whereas 7 (18.4%) cases had a bilateral presentation. Visual acuity of stromal HSV keratitis patients after 1-month follow-up (Table 3).

**Table 3:** Visual acuity of stromal HSV keratitis patients after 1 month (n= 38).

Visual acuity at presentation	n (%)
6/6-6/18	9 (23.68)
6/24-6/60	19 (50.00)
<6/60-3/60	7 (18.42)
<3/60	3 (7.80)

## DISCUSSION

Our study showed the prevalence of stromal keratitis to be 33.93% among Herpes Simplex Keratitis (HSK) patients. The stromal keratitis patients' mean age of presentation was of 47.19±19.14 years which is similar to other studies.^7^ In the subcontinent, a study in India reported a mean age of 29.9±16.69 years for females and 32.09±15.79 years for males, and a similar in Nepal while the prevalence of stromal keratitis was higher and lower respectively.^5,8^ This signifies that HSK affects largely the productive age group, and hence any significant scarring can have an adverse effect on this particular population.

In our study among the total 38 cases of stromal keratitis, 28 were female and 10 were male, whereas other studies done in the Indian subcontinent have shown a clear male preponderance for HSK.^5,9^ The studies done earlier showed a lower incidence of stromal involvement whereas in our study stromal involvement has also increased which points toward a shift in natural history.^8,10,11^

The common symptoms presented were 22 patients presented with mild pain, 90 with redness, 49 photophobia, 53 with decreased vision and 44 with watery discharge which is similar to other studies.^5,9,12^ However, blurring of vision as a less frequent symptom which may have been due to the fact that that study reported a very lower number of stromal diseases and visual disturbance mostly being attributed to stromal involvement.^10^ Most of the patients 50% had history of spontaneous onset of disease followed by other triggering factors i.e. 26.3%stress, 34.2% fever, 21.05% foreign body, 15.7% skin eruptions and 13.1% UV light. Another study has showed the most common triggering factor was stress 15.6% followed by fever and trauma 11.1%.^8^

In our study vision at presentation, 13.15% had a 6/66/18, 31.5% had a vision of 6/24-6/60, 34.21% less than 6/60-3/60, and 21.05% <3/60 which is similar to other studies.^7^ The visual acuity in different types of keratitis showed the decrease in vision with the involvement of stroma and endothelium. In the present study, the extent of visual gain at one month follow up were 23.68% had visual acuity of 6/6-6/18, 50% had 6/246/60, 18.42% <6/60-3/60, and 7.8% <3/60 which is similar to another study.^2^

This study was conducted in one tertiary eye hospital, so these results cannot be generalized to the whole country also the study had convenience sampling, and there could have been selection bias in the selection of cases.

## CONCLUSIONS

The prevalence of stromal keratitis among herpes simplex keratitis was similar to studies conducted in similar settings. A further large-scale and prospective study needs to be conducted to know the disease pattern of Herpes simplex keratitis in a developing country.
